# Experimental and DFT data of *p*-chlorocalix[4]arene as drugs receptor

**DOI:** 10.1016/j.dib.2020.106263

**Published:** 2020-09-02

**Authors:** M.A. Kadir, F.I. Abdul Razak, N.S.H. Haris

**Affiliations:** a Advanced Nano Materials (ANoMa) Research Group, Faculty of Science and Marine Environment, Universiti Malaysia Terengganu, 21030 Kuala Terengganu, Terengganu, Malaysia; b Faculty of Science and Marine Environment, Universiti Malaysia Terengganu, 21030 Kuala Terengganu, Terengganu, Malaysia; c Department of Chemistry, Faculty of Science, Universiti Teknologi Malaysia, 81310 UTM Johor Bahru, Johor, Malaysia

**Keywords:** *p*-Chlorocalix[4]arene, Paracetamol, Interaction energy, Molecular receptors, Gaussian

## Abstract

The data in this article provide information on spectroscopic and theoretical data for *p*-chlorocalix[4]arene when combined with selected drugs, such as paracetamol, ibuprofen, and cetirizine. The present spectroscopic data are generated from Fourier Transform Infrared (FTIR), Nuclear Magnetic Resonance (^1^H NMR and ^13^C NMR), and Ultraviolet-Visible spectroscopy (UV-Vis) as the key tools for molecular characterization. The measurement of the optimization energy, interaction energy, and the band gap energy between the molecules was calculated by Gaussian 09 software. It is interesting to note that of the three titled drugs identified, *p*-chlorocalix[4]arene showed the highest interaction energy with paracetamol, followed by ibuprofen and cetirizine.

## Specifications Table

**Table:** 

Subject	Chemistry
Specific subject area	Synthetic chemistry, spectroscopy, computational chemistry
Type of data	Table Figure
How data were acquired	FTIR Perkin Elmer Spectrum 100 and the spectra were recorded in the range of 4000-400 cm^−1^ utilizing potassium bromide (KBr) pellet, UV-Vis (Spectrophotometer Shimadzu UV-1800), ^1^H, and ^13^C NMR spectra analyzed by Bruker Avance III 400 spectrometer. Computational calculations were performed using Gaussian 09 software package on the desktop CPU@ 3.10GHz processor provided by Faculty of Science, UTM, 64-bit operating system with an x64-based processor, a 4.00 installed memory and 3.87GB RAM.
Data format	Raw and analyzed
Parameters for data collection	The reaction mixture is heated up to 70°C until completion. The computational data are calculated at ground state and TD-SCF/DFT/B3LYP/6-31G.
Description of data collection	The compound was characterized by a spectroscopic method and the application was observed by a UV spectrophotometer. Gaussian 09 software was used to calculate the interactions between host (*p*-chlorocalix[4]arene) and guest molecules (paracetamol, ibuprofen, and cetirizine)
Data source location	Faculty of Science and Marine Environment, Universiti Malaysia Terengganu, 21030 Kuala Terengganu, Terengganu, Malaysia. Type of sample: Experimental data Department of Chemistry, Faculty of Science, Universiti Teknologi Malaysia, 81310 UTM Johor Bahru, Johor, Malaysia Type of sample: Computational data
Data accessibility	Data is included in this article

## Value of the Data

• The dataset in this study can be used as a reference and guidance for further investigation of calix[4]arene derivatives as a drug-receptor.
• The discovery of new methods in computational chemistry offers research opportunities and further collaborations with other researchers who are interested in the host-guest chemistry of Calix molecules.
• Since the outbreak of pandemic Covid-19, computational modelling has been utilized in explaining the chemical interactions between molecules. This study is an example of a contribution made by DFT studies in enhancing understanding of molecular interactions between the host and guest molecules.

## 1 Data Description

**Fig. 1:**
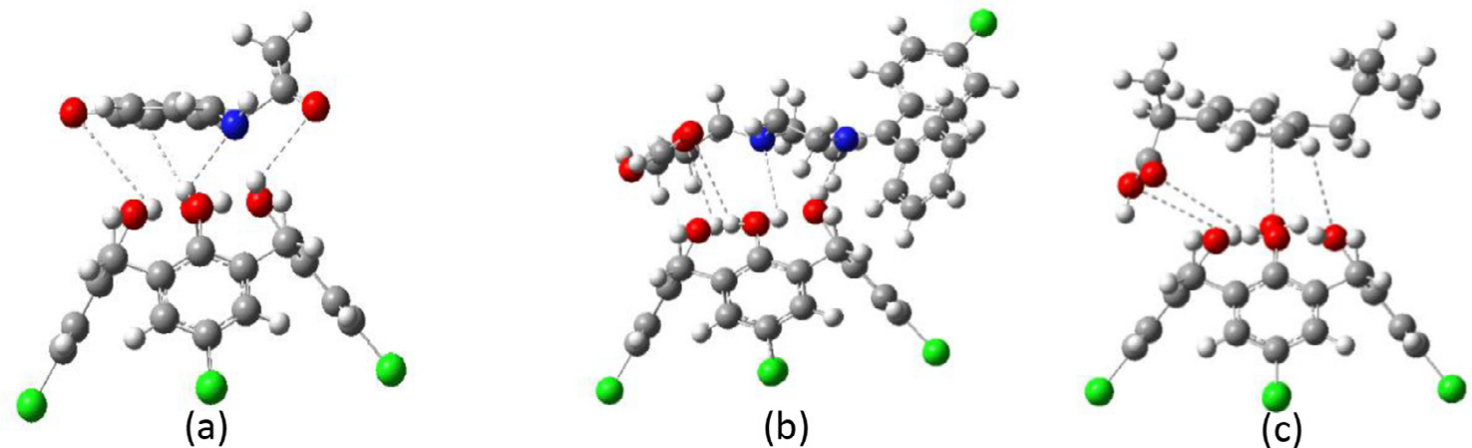
Hydrogen bonding and π-π interaction between *p*-chloro[4]arene with (a) paracetamol (b) ibuprofen and (c) cetirizine.

Compound *p*-chlorocalix[4]arene was prepared from the reaction between calix[4]arene, ferric chloride, and thionyl chloride in tetrachloromethane [1]. The title compound was obtained as a brown solid after tetrachloromethane was removed under vacuum, and was further characterized by Fourier Transform Infrared (FTIR), Nuclear Magnetic Resonance (^1^H NMR and ^13^C NMR) and Ultraviolet-Visible spectroscopy (UV-Vis). Fig. 1 shows the illustration of computer modelling using Gaussian 09 software. The *p*-chlorocalix[4]arene acted as molecular host, and three selected guest drugs namely paracetamol, ibuprofen, and cetirizine, acted as the guest molecules. The result showed that both hydrogen bonding and π-π interactions combined to stabilize the molecular structures [2]. Theoretical models proposed by Gaussian were applied to measure the optimization energy, binding energy, and interaction energy of *p*-chlorocalix[4]arene with the drugs. The calculated data are listed in Table 1.

**Table 1:** Calculated theoretical data for corrected binding energy and interaction energy between *p*-chlorocalix[4]arene with paracetamol, ibuprofen, and cetirizine.

Set No.	Optimization energy, kJ/mol	ΔE_Binding_ (corrected), kJ/mol	ΔE_Interaction_ (corrected), kJ/mol
*p*-chlorocalix[4]arene	-8456188.89	-	-
cetirizine	-4227179.38	-	-
ibuprofen	-1724255.82	-	-
paracetamol	-1353431.97	-	-
*p*-chlorocalix[4]arene + cetirizine	-12683393.03	-4.72174264	24.60728374
*p*-chlorocalix[4]arene + ibuprofen	-10180464.16	3.19451973	25.86844207
*p*-chlorocalix[4]arene + paracetamol	-9809665.04	-16.67581839	76.12587387

**Fig. 2:**
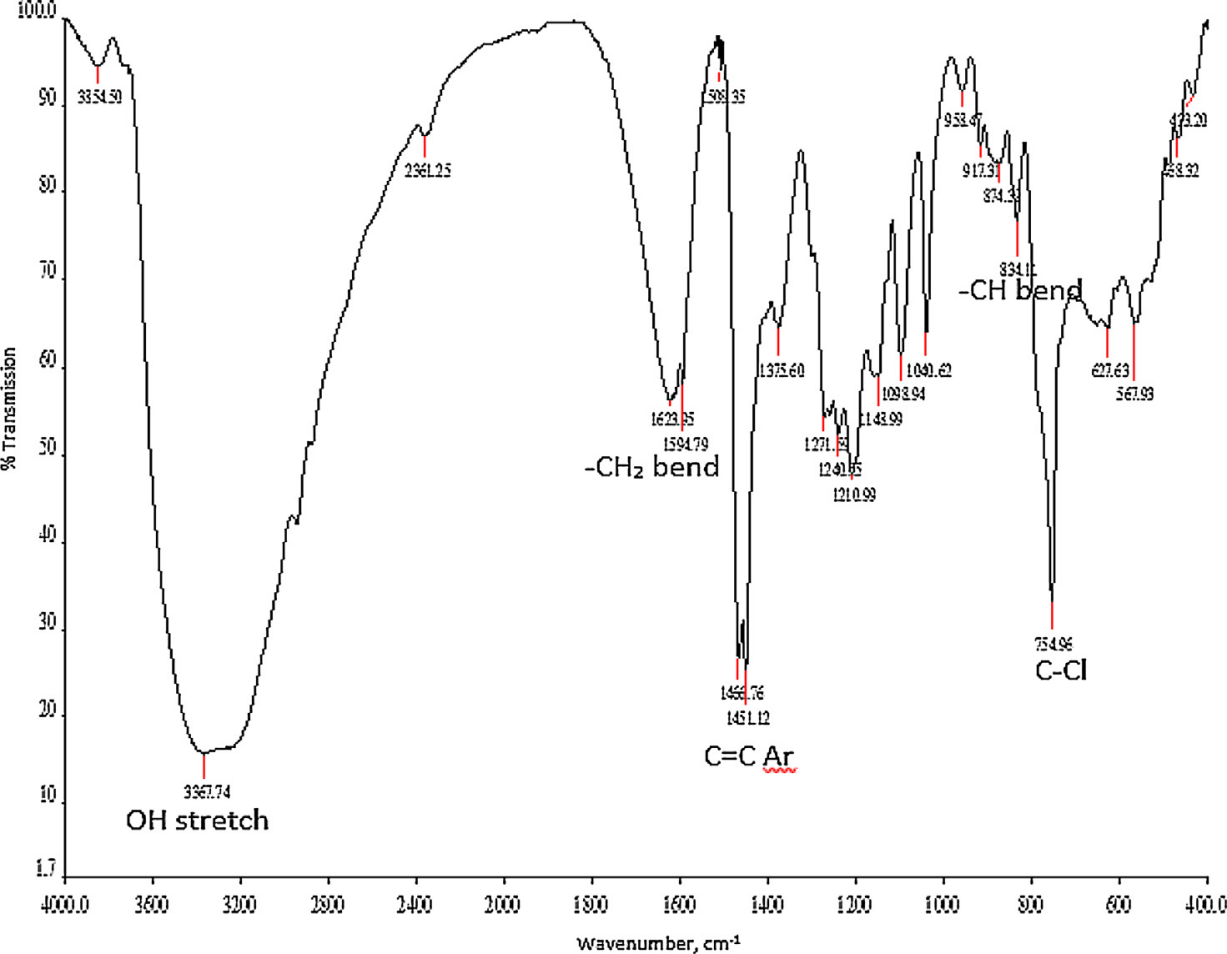
The FTIR spectrum of *p*-chlorocalix[4]arene.

**Fig. 3:**
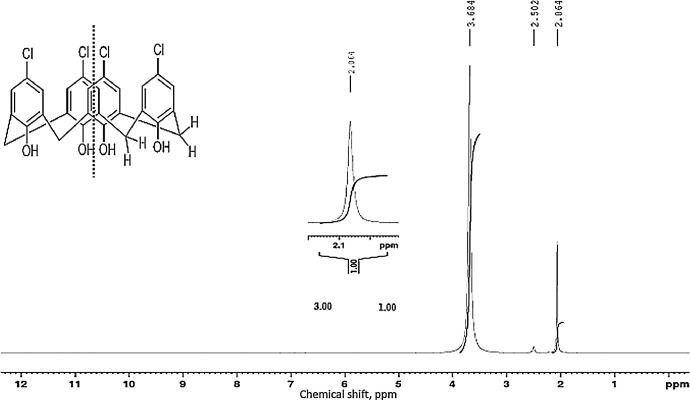
^1^H NMR of *p*-chlorocalix[4]arene.

**Fig. 4:**
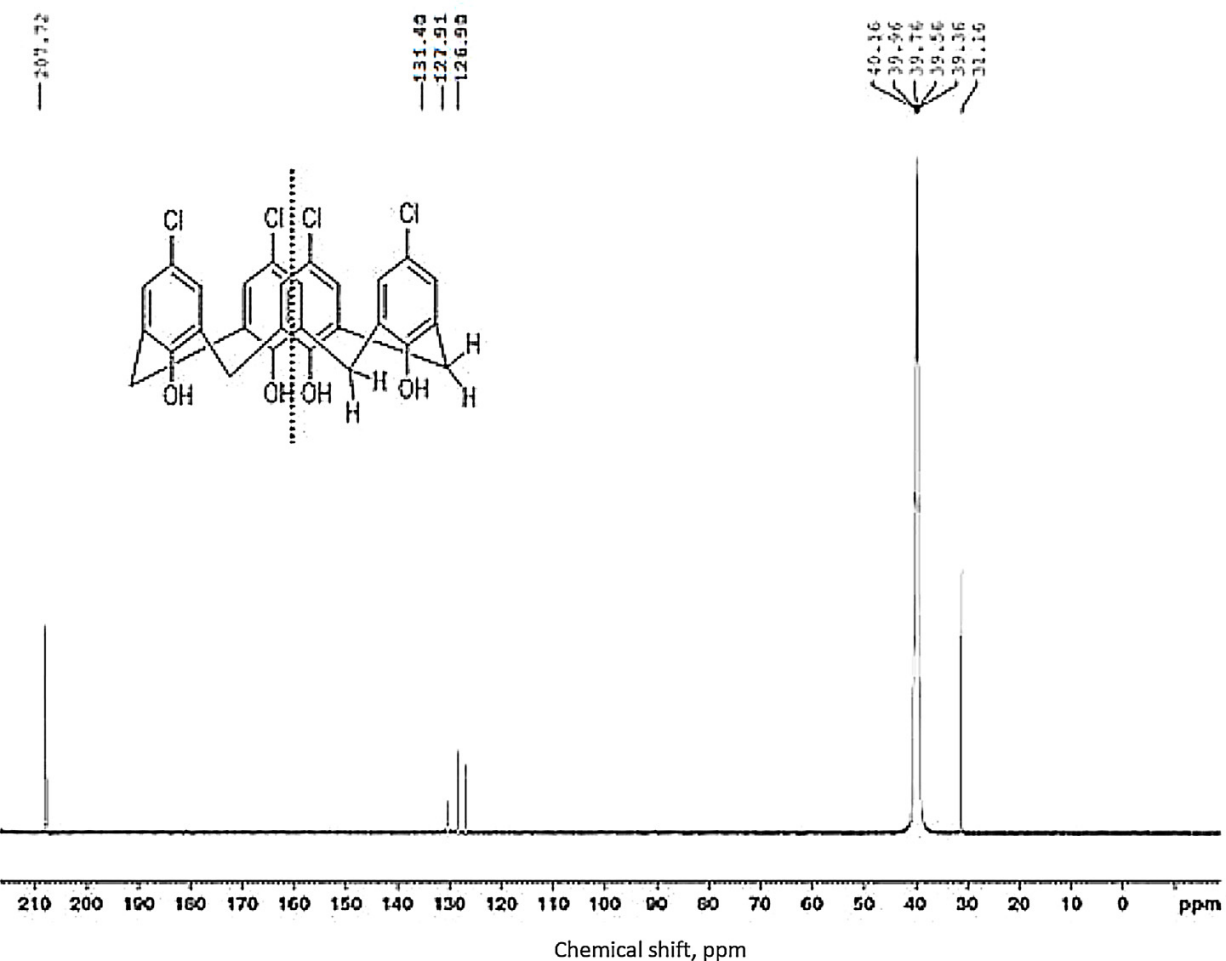
^13^C NMR of *p*-chlorocalix[4]arene.

**Fig. 5:**
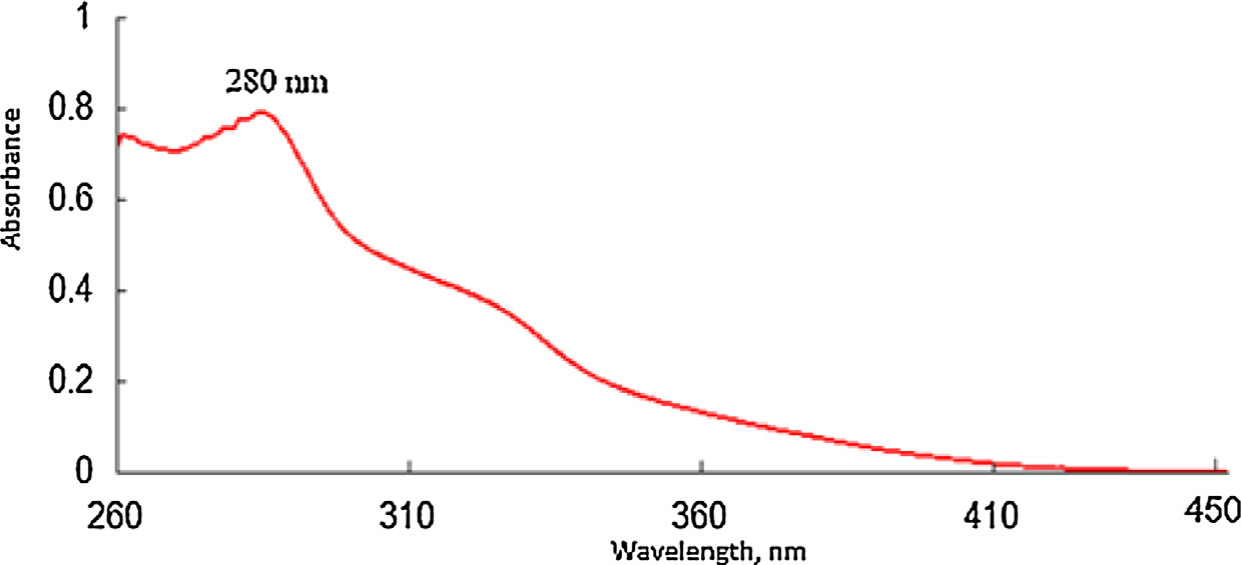
UV-Vis of *p*-chlorocalix[4]arene.

**Fig. 6:**
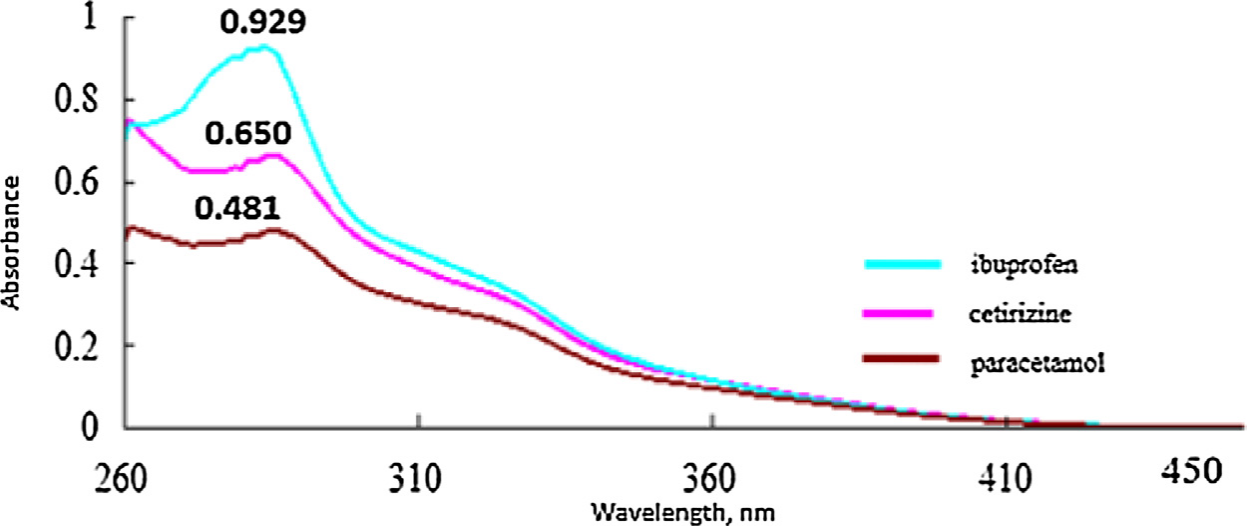
UV-Vis spectrum of *p*-chlorocalix[4]arene with paracetamol, cetirizine, and ibuprofen.

The descriptive measurement showed that strong interaction occurs between *p*-chlorocalix[4]arene with paracetamol (76.12587387 kJ/mol), which reflects a higher affinity of the interaction between both molecules [3] as compared to ibuprofen (25.86844207 kJ/mol) and cetirizine (24.60728374 kJ/mol). This finding is in line with previous study that confirmed the interaction energy is in the range for a non-covalent interaction [4]. Spectroscopic data from FTIR, NMR, and UV-Vis are depicted in Figs. 2, Fig. 3, Fig. 4 and –Fig. 5, with the data tabulated in Tables 2 and 3, respectively. Fig. 6 indicates that *p*-chlorocalix[4]arene has the highest binding interaction with paracetamol as compared to ibuprofen and cetirizine, where the absorption peaks appeared at the lowest absorbance in the UV spectrum (0.481, 280 nm).

**Table 2:** FTIR data for *p*-chlorocalix[4]arene.

Types of Vibration	Wavenumber (cm^−1^)
OH	3367
C=C Ar	1594
-CH_2_ bend	1466
-CH bend	834
C-Cl	754

**Table 3:** Data analysis of ^1^H & ^13^C NMR of *p*-chlorocalix[4]arene.

Chemical shift, δ_H_, ppm	Multiplicity
2.06	8H, s, δ, (CH_2_)
3.68	8H, s, δ, (benzene-CH)

Chemical Shift, δ_C_, ppm	Type of Carbons
31.16	-CH_2_
126.90	py-Cl
127.91, 131.40	py(C=C)
207.72	C-OH

**Fig. 7:**
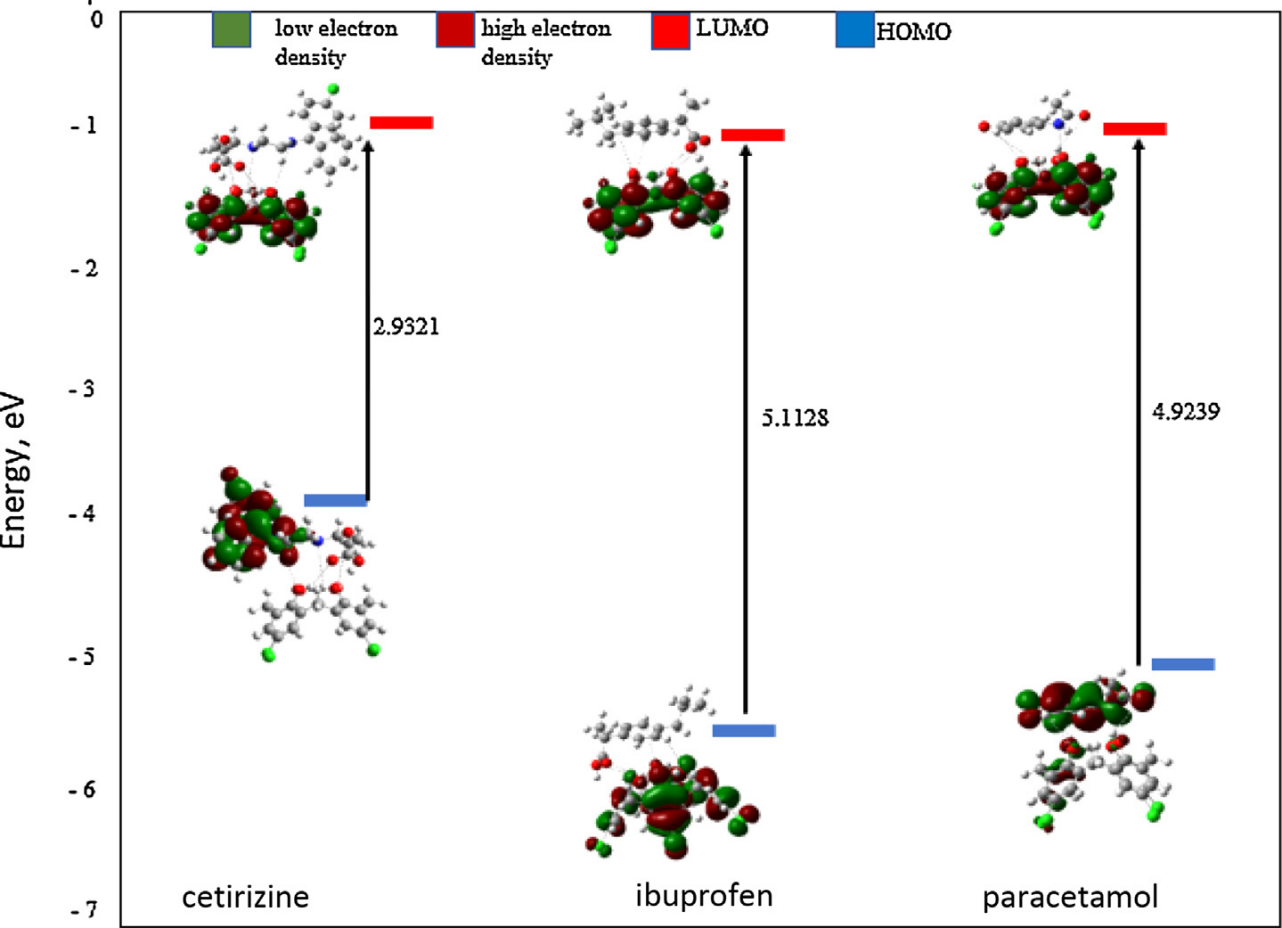
Schematic energy level diagram of molecular orbital of *p*-chlorocalix[4]arene.

The molecular orbital (MO) diagram of *p*-chlorocalix[4]arene and the three title drugs are presented in Fig. 7 to elucidate the electronic transition within the complex. The diagram showed that *p*-chlorocalix[4]arene and cetirizine has the lowest energy band gap (2.9321 eV) as compared to paracetamol (4.9239 eV) and ibuprofen (5.1128 eV). In theory, when conjugation increases, the band gap between HOMO-LUMO decreases, thus less energy is required for the electron to excite from HOMO to LUMO [5]. This is applied to cetirizine because this compound exhibits more conjugated electrons when compared to paracetamol and ibuprofen.

## 2 Experimental Design, Materials and Methods

### 2.1 Synthesis of p-chlorocalix[4]arene

Synthesis of *p*-chlorocalix[4]arene was carried out accordingly to the literature procedure with some modifications [1]. Calix[4]arene (1.0 g) was dissolved in tetrachloromethane, CCl_4_ (50 mL). The mixture was left to stir before iron (III) chloride, then the FeCl_3_ (0.19 g) was added. After that, thionyl chloride, SOCl_2_ (0.64 mL) was added dropwise and heated at 70°C for 24 hours. Upon completion, the solvent was removed using a rotatory evaporator to give a brown solid. The residue was dissolved in 75 mL of dichloromethane and washed with 50 mL of saturated sodium bicarbonate two times. Dichloromethane was dried over magnesium sulfate and the solvent was removed by using a rotatory evaporator to form the product as brown pale solid in 70% yield.

### 2.2 Preliminary UV-Vis studies

The *p*-chlorocalix[4]arene (0.56 mg, 0.001 mmol) was prepared in dimethyl sulfoxide (10 mL). The solution of analytes was also prepared by dissolving paracetamol (0.151 mg, 0.001 mmol), ibuprofen (0.206 mg, 0.001 mmol) and cetirizine (0.30 mg, 0.001 mmol) in dimethyl sulfoxide (10 mL). The solution was mixed and the obtaining spectrum was observed for any changes at 280 nm.

### 2.3 Computational method

All compounds were calculated using Density Functional Theory (DFT)/B3LYP method with a 6-31G (d,p) basis set. The geometry was optimized without any symmetry constraints and performed at the gas phase. The value formula of uncorrected binding energy was collected from optimization calculation, as displayed in Eq. (1). The collected data of optimization energy was essential to obtain the corrected binding energy [6] by adding the Basis Set Superposition Error (BSSE) value. The correction value is required to remove the artificial energy lowering caused by the BSSE [7]. The formula of corrected binding energy was displayed in Eq. (2). Meanwhile, the value of interaction energy was collected from interaction energy calculation, the formula was displayed in Eq. (3). The data of Table 1 can be used [3,4] as a guideline for further interpretation. The HOMO and LUMO computational analysis have been used to clarify the electronic transition within the complex by TD-SCF/DFT/B3LYP method with a 6-31G (d,p) basis set in a solvent phase, where DMSO was used as a solvent match for the experimental method.

(1) $$\Delta E_{Binding}\left(\text{uncorrected}\right),\;kJ/mol=\Delta E_{complex}-\left(\Delta E_{calix}+\Delta E_{drug}\right)$$

(2) $$\Delta E_{\text{Binding}}\left(\text{corrected}\right),\text{kJ}/\text{mol}=\Delta E_{\text{Binding}}\left(\text{uncorrected}\right)+\Delta E_{\text{BSSE}}$$

(3) $$\Delta E_{\text{Interaction}}\left(\text{corrected}\right),\text{kJ}/\text{mol}=\Delta E_{\text{Interaction}}\left(\text{uncorrected}\right)+\Delta E_{\text{BSSE}}$$

## Declaration of Competing Interest

The authors declare that they have no known competing financial interests or personal relationships which have, or could be perceived to have, influenced the work reported in this article.

## Appendix Supplementary materials

Image, application 1
